# Ordered Disorder of the Astrocytic Dystrophin-Associated Protein Complex in the Norm and Pathology

**DOI:** 10.1371/journal.pone.0073476

**Published:** 2013-08-27

**Authors:** Insung Na, Derek Redmon, Markus Kopa, Yiru Qin, Bin Xue, Vladimir N. Uversky

**Affiliations:** 1 Department of Molecular Medicine, Morsani College of Medicine, University of South Florida, Tampa, Florida, United States of America; 2 Institute for Biological Instrumentation, Russian Academy of Sciences, Pushchino, Moscow Region, Russia; University of Alberta, Canada

## Abstract

The abundance and potential functional roles of intrinsically disordered regions in aquaporin-4, Kir4.1, a dystrophin isoforms Dp71, α-1 syntrophin, and α-dystrobrevin; i.e., proteins constituting the functional core of the astrocytic dystrophin-associated protein complex (DAPC), are analyzed by a wealth of computational tools. The correlation between protein intrinsic disorder, single nucleotide polymorphisms (SNPs) and protein function is also studied together with the peculiarities of structural and functional conservation of these proteins. Our study revealed that the DAPC members are typical hybrid proteins that contain both ordered and intrinsically disordered regions. Both ordered and disordered regions are important for the stabilization of this complex. Many disordered binding regions of these five proteins are highly conserved among vertebrates. Conserved eukaryotic linear motifs and molecular recognition features found in the disordered regions of five protein constituting DAPC likely enhance protein-protein interactions that are required for the cellular functions of this complex. Curiously, the disorder-based binding regions are rarely affected by SNPs suggesting that these regions are crucial for the biological functions of their corresponding proteins.

## Introduction

It is recognized now that many biologically active proteins, known as intrinsically disordered proteins (IDPs), lack stable tertiary and/or secondary structure under physiological conditions *in vitro* [1]–[34]. They are highly abundant in nature, with ∼25–30% of eukaryotic proteins being mostly disordered, and with >50% of eukaryotic proteins and >70% of signaling proteins having long IDP regions (IDPRs) [35]–[39]. IDPs possess remarkable structural heterogeneity, ranging from completely structure-less, coil-like conformational ensembles to compact molten globule-like structural ensembles [33], [40]–[42]. Furthermore, disorder can affect proteins to a different degree, and some proteins are disordered as a whole, whereas other proteins possess a mosaic or hybrid structure containing both ordered and disordered regions [33], [40]–[43]. Functional repertoire of IDPs/IDPRs is very broad and complements functions of ordered proteins and domains. Disorder-based functions may arise from the specific disorder form, from inter-conversion of disordered forms, or from transitions between disordered and ordered conformations [3], [4], [9], [10], [33]. Many IDPs/IDPRs possess an exceptional binding promiscuity often associated with the ability to fold in a template dependent manner, where a single IDPR can bind to multiple partners gaining very different structures in the bound state [28], [44]. Often, IDPs are involved in regulation, signaling and control pathways, where binding to multiple partners and high-specificity/low-affinity interactions play a crucial role and where IDPs/IDPRs play different roles in regulation of the function of their binding partners and in promotion of the assembly of supra-molecular complexes [1], [3], [5]–[7], [14], [15], [19], [24]–[28], [33].

IDPs and IDPRs are the key players in various protein-protein interaction networks, being especially abundant among hub proteins and their binding partners [14], [45]–[49]. Furthermore, regions of pre-mRNA which undergo alternative splicing commonly encode for the IDRs [50]. This association of alternative splicing and intrinsic disorder helps proteins to avoid folding difficulties and provides a novel mechanism for developing tissue-specific protein interaction networks [32], [50].

Since the absence of rigid structure in IDPs is encoded in the specific features of their amino acid sequences [2], [4], [9], [10], [33], [51], multiple computational tools were elaborated for evaluation of the abundance of intrinsic disorder in proteins and proteomes, for the analysis of the peculiarities of disorder distribution within a given protein, and for finding disorder-based functional sites [16], [52]–[61]. Multifactorial computational studies indicated the abundance and functional importance of intrinsic disorder in various proteinaceous machines, such as nucleosome [62], spliceosome [63], [64], ribosome [65], nuclear pore [66]–[68], the mediator complex [69], and many transcription-related complexes [70]. Computational analysis of the transmembrane proteins revealed that the intracellular regions of single-path proteins are heavily enriched in disorder [71], [72], that the cytoplasmic signaling domains of various cell receptors are frequently disordered [73], that many transmembrane and peripheral membrane proteins contain disorder-based binding sites known as molecular recognition features [74], that the majority of human plasma membrane proteins contain long disordered regions [75], and that the IDPRs from helical bundle integral membrane proteins, those from β-barrel integral membrane proteins, and IDPRs from water soluble proteins all exhibit statistically distinct amino acid compositional biases [76]. Although the multifarious functional roles of disorder in nuclear pore were reported [67], [68], [77], no other membrane-associated protein complexes were subjected to the detailed analysis focused at protein intrinsic disorder. To fill this gap, we report here a computational study on the abundance and roles of intrinsic disorder in five proteins (aquaporin-4, Kir4.1, Dp71, α-1 syntrophin, and α-dystrobrevin) that constitutes the membrane-bound astrocytic dystrophin-associated protein complex (DAPC).

**Figure 1:**
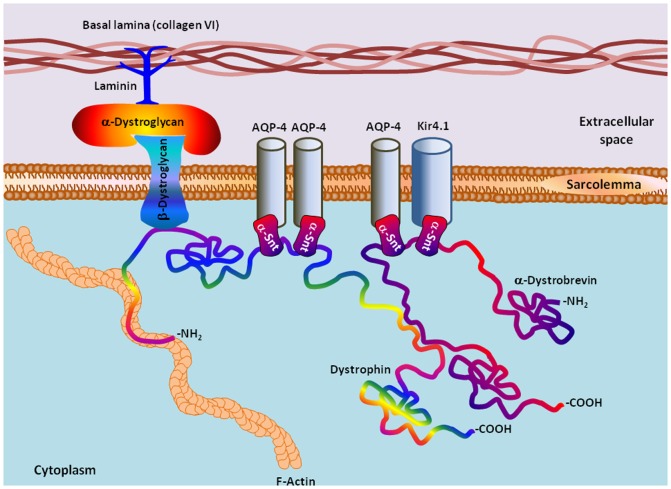
Schematic representation of the astrocytic DAPC analyzed in this study. The major focus of our work was dystrophin (Dp71) and the Dp71-associated proteins AQP-4, Kir4.1, α-1 syntrophin (α-Snt), and α-dystrobrevin.

The major function of dystrophin is to anchor the extracellular matrix to the cytoskeleton via the F-actin. In a classic view, dystrophin of skeletal and cardiac muscle associates with various proteins to form the dystrophin-associated protein complex (DAPC) [78]. However, DAPC is also positioned in the extracellular membrane of the astrocytic endfeet abutting the blood vessels [79], and it can be found at the neuromuscular junction (NMJ) and at a variety of synapses in the peripheral and central nervous systems where it has a structural function in stabilizing the sarcolemma [80]. The core of the astrocytic DAPC is composed of seven proteins (see Figure 1), an extracellular peripheral glycoprotein α-dystroglycan, a transmembrane protein β- dystroglycan, two specific transmembrane channels, aquaporin-4 (AQP-4) and Kir4.1, and three cytosolically located proteins, such as a fifth isoform of dystrophin (Dp71), α-1 syntrophin (SNTA1), and α-dystrobrevin (DTN-A, also known as dystrophin-related protein 3)) [78], [81]. The heterodimer of the α- and β-dystroglycans is the central DAPC components, where the α-dystroglycan interacts with the laminins in the basal lamina, and the transmembrane β-dystroglycan is involved in connecting the extracellular matrix to the cytoskeleton. The AQP-4 is an extremely important channel maintaining the osmotic balance of the blood-brain barrier (BBB) [82], whereas Kir4.1 acts as an inwardly rectifying K^+^ channel that has a role in potassium buffering [83]. The PDZ and SU domains of the α-1 syntrophin and other syntrophins are responsible for a set of the cross-protein contacts, interacting with AQP-4, Kir4.1, Dp71 and dystrobrevin-α [78]. A dystrophin isoform Dp71 is reported as an anchoring protein of AQP-4 and Kir4.1 [84], [85]. α-Dystrobrevin is another DAPC component which connects proteins related to the complex in a fashion similar to Dp71 [78]. The structural and functional relationship between each of the members of the DAPC has been known for some time [78], [81] (Figure 1). Since this study is focused on AQP-4, Kir4.1, Dp71, α-1 syntrophin, and α-dystrobrevin, these important DAPC components are briefly introduced below.

The AQP-4 protein is associated with a number of human diseases, but most importantly with Neuromyelitits Optica (NMO). Also referred to as Devic's disease, NMO is an inflammatory demyelinating disease that selectively affects optic nerves and spinal cord. In 2004, an Anti-AQP-4 antibody was discovered that had a significant impact on the diagnosis and understanding of the molecular NMO mechanisms. The discovery of an NMO disease-specific antibody, NMO-IgG [86], was fueled by the observation that immunoglobulin and complement deposition in active lesions followed a distinct rim and rosette vasculocentric pattern, suggesting an antibody-mediated mechanism of disease [86]. Later, the AQP-4 protein was identified as the NMO-IgG target [87].

Potassium channels play an important role in the DAPC signal transduction pathways suggesting that AQP-4 and potassium channels, such as Kir4.1, might work together [83]. Here, AQP-4 likely acts in concert with potassium and bicarbonate channels to regulate water dynamics in the central nervous system (CNS) between the brain, blood, and cerebrospinal fluid (CSF). Overall, this cooperation plays an integral role in maintaining both brain fluid volume and ion homeostasis [88], [89]. The clinical manifestations of NMO are comparable to those found in AQP-4 knockout mice, which implicates a correlation between AQP-4 and Kir4.1. In support of this implication is the subcellular co-localization of AQP-4 with the inwardly rectifying potassium channel Kir4.1 [90]. These results support the hypothesis that AQP-4 and Kir4.1 interact with each other [88]–[90].

The ATP-sensitive, inwardly rectifying potassium channel Kir4.1 is a protein that is encoded by the *KCNJ-10* gene in humans [91]. It has the tendency to allow a greater extent of potassium to flow into the cell, rather than out. Potassium channels are found in various parts of the human body, but most notably in cardiac, liver, endothelial and neuronal cells. By carefully modulating the net potassium currents, Kir4.1 helps to maintain a resting membrane potential. The basic building blocks of the Kir4.1 channel are two transmembrane helices with cytoplasmic N- and C-termini and an extracellular loop which folds back to form the pore-lining ion selectivity filter [92]. Mutations of Kir4.1 can cause a number of symptoms including Epilepsy, Ataxia, Sensorineural deafness and Tubulopathy; these are collectively known East syndrome [93].

The next protein in the study is dystrophin, which is a product of the Duchenne Muscular Dystrophy (*DMD*) gene. More specifically we looked at the fifth isoform of dystrophin, known as Dp71 that constitutes the C-terminal domain of the dystrophin protein. *DMD* being one of the longest human genes, with a total length of ∼2.2 megabases, is located on the X chromosome. In its mutant form, it is responsible for various myopathies [94]–[96], including Duchenne muscular dystrophy [97], Becker muscular dystrophy [98], X-linked dilated cardiomyopathy [99], and sporadic dilated cardiomyopathy [100]. Dystrophin is normally present within the sarcolemma of the skeletal muscle as part of a large protein complex which forms a linkage between the cytoskeleton, the sarcolemma, and the extracellular matrix [101]. The *DMD* gene exhibits complex transcriptional regulation and drives the synthesis of a variety of dystrophin isoforms through utilization of different promoters. Full-length dystrophin (427 kDa) is derived from three independent promoters, located at the 5′-end of the *DMD* gene, that regulate its spatiotemporal expression in muscles, brain structures, and cell types [102]–[104].

Another important DAPC protein is α-1 syntrophin. It is encoded by the *SNTA1* gene and acts as an adapter between the AQP-4 and Dp71 proteins by binding via its PDZ domain to the AQP-4 C-terminus. The PDZ domain of α-1 syntrophin, which is the most abundant gene product in the heart, has been reported to bind to the C-terminal domain of the cardiac voltage-gated sodium channels (SkM2) causing alterations of the ion channel activity which causes long QT syndrome [105], [106]. Long QT syndrome (LQTS) is an inborn, abnormal heart rhythm condition characterized by a delayed repolarization of the cardiac muscle, and can lead to dangerous episodes of arrhythmias, cardiac arrest, or even sudden death. LQTS can arise from mutations of one of several associated genes [105], and is inherited in either an autosomal dominant or autosomal recessive manner.

The last protein considered in this study is dystrobrevin, which can bind to other molecules in the multi-protein DAPC. Although there are two isoforms dystrobrevin are known, the α- and the β-dystrobrevins, the focus of our research was on the α-isoform because of its interaction with syntrophin and Dp71. In fact, based on the yeast two-hybrid (Y2H) analysis it has been concluded that α-dystrobrevin is involved in the direct heterodimerization with the dystrophin [107]. This is based on findings that suggest the C-terminus of dystrobrevin binds to the C-terminus of dystrophin. The gene that codes for α-dystrobrevin is called *DTNA*. Mutations in *DTNA* are known to cause the left ventricular non-compaction type 1 (LVNC1) disease, which is defined by the presence of poor systolic function, and is sometimes associated with other cardiac abnormalities including atrial or ventricular septal defects [108].

In the overall analysis of the DAPC, we found that multiple proteins play significant roles in the formation of the complete, functional complex. Although many of their roles have yet to be elucidated, the physical interactions between these proteins have been studied in detail. Among other analyses, we looked for SNPs in regions that were specifically involved in the interactions between the DAPC proteins. We also looked at the entire amino acid sequences of these proteins to see where the most frequent single nucleotide polymorphisms (SNPs) occurred. In this way we wanted to connect the presence and occurrence of specific SNPs with the destabilization of the DAPC and also to find a potential correlation between such DAPC destabilization and the presence and/or duration of the epileptic seizures in humans. Through our study we have come to hypothesize that the amino acid composition, as well as mutations and variations within each protein's amino acid sequence, affects the DAPC stability and therefore function. To verify this hypothesis, we identified the conserved amino acid sequences within the binding sites of the protein-protein interactions, as well as any mutations, single nucleotide polymorphisms (SNPs), and intrinsically disordered protein regions (IDPRs) related to the DAPC proteins. According to an earlier study emphasizing that the conserved motifs in IDPR have serious impacts on protein function [109], we performed IDPR analysis of the important binding sites in each protein in order to find the conserved amino acid motifs. We also analyzed all available disease mutation and SNP data to cross check the effects that these types of variations have on the DAPC functions.

## Results and Discussion

### Intrinsic Disorder and Its Conservation within the DAPC Members

**Figure 2:**
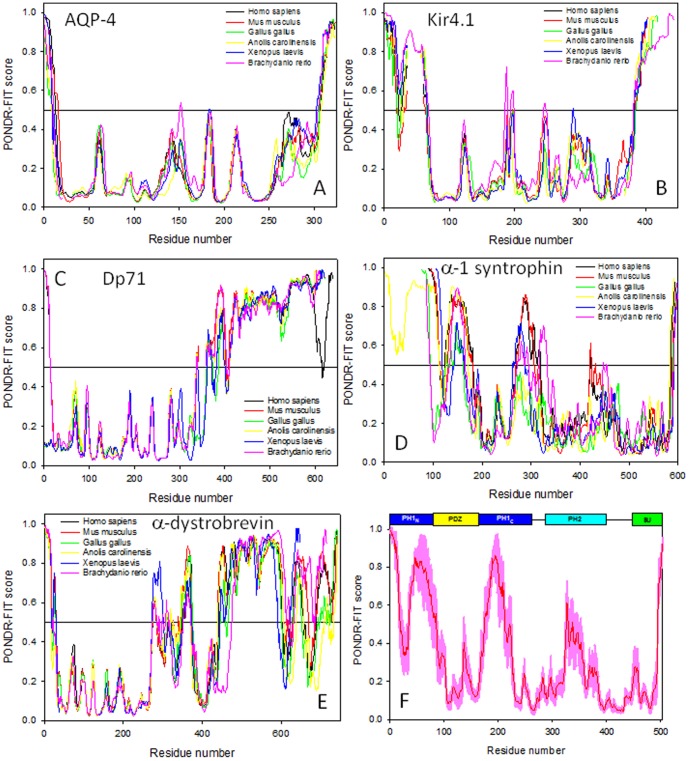
Abundance of intrinsic disorder in proteins from six vertebrate DAPCs. **A**. AQP-4; **B**. Kir4.1; **C**. Dp71; **D**. α-1 syntrophin; and **E**. α-dystrobrevin. PONDR-FIT scores are shown for corresponding proteins from *Homo sapiens* (black lines), *Mus musculus* (red lines), *Gallus gallus* (green lines), *Anolis carolinensis* (yellow lines), *Xenopus laevis* (blue lines), and *Brachydanio rerio* (pink lines). Disorder profiles were manually aligned by visual inspection to ensure matching of the most characteristic features. The number of gaps introduced in affected proteins during these visual alignments was kept to a minimum. Plot **F** represents a PONDR-FIT plot for human α-1 syntrophin. Domain structure of this protein is also indicated in relation to the disorder profile. Pink shadow around PONDR-FIT curve represents distribution of errors in the evaluation of the PONDR-FIT scores.

To understand the peculiarities of intrinsic disorder predispositions of AQP-4, Kir4.1, Dp71, α-1 syntrophin, and α-dystrobrevin, we investigated the distributions of the predicted disorder propensity (in a form of the plots showing the per-residue PONDR-FIT scores produced by the PONDR-FIT disorder prediction algorithm) in these five DAPC proteins from selected vertebrates, such as mammals (*Homo sapiens* and *Mus musculus*), bird (*Gallus gallus*), reptile (*Anolis carolinensis*), amphibian (*Xenopus laevis*), and fish (*Brachydanio rerio*). Table 1 lists the analyzed proteins together with their corresponding UniProt IDs (www.UniProt.org). Figure 2 represents the results of this analysis and shows that all five proteins from all the organisms analyzed in this study are typical hybrid proteins [43]; i.e., proteins possessing both ordered domains and variously disordered regions. Figure 2 shows that AQP-4 and Kir4.1 have similar disorder profiles with areas of high disorder at the N- and C-termini, and with the majority of sequences having disorder values between the 0.1 and 0.5, although in both proteins, there are a few areas which approach the 0.5 disorder threshold. The low abundance of disorder in AQP-4 and Kir4.1 is rather typical for the transmembrane proteins [76]. Also, disordered tails of transmembrane channels are common points of channel regulation and interaction with other proteins. The disorder profile of Dp71 is quite different from the two transmembrane proteins. Though its N- and C-termini are highly disordered, as seems to be a common feature for all five proteins of interest, the Dp71 protein seems to be evenly split between ordered and disordered domains, with the first half of the protein falling in the 0.1–0.3 disorder range, while the second half falling in the 0.7–0.9 disorder range. Of course, the protein has little “spikes” of disorder in its area of order, and order in its area of disorder. Finally, the disorder profiles of α-1 syntrophin and α-dystrobrevin possess somewhat more sporadic appearance containing multiple clusters of order and disorder.

**Table 1:** Members of the dystrophin-associated protein complex (DAPC) analyzed in this study.

	*Homo sapiens*	*Mus musculus*	*Gallus gallus*	*Anolis carolinensis*	*Xenopus laevis*	*Brachydanio rerio*
Aquaporin-4	P55087	P55088	F1NJZ6*	G1KAC7*	F6Z564*	F1R274*
Kir4.1	P78508	Q9JM63	F1P0R9*	G1KIK1*	F6PWL6*	E7FD27*
Dp71	P11532-5	A2A9Z1*	F1NS97*	G1KGB9*	F7BS74*	F1QDK4*
α-1 syntrophin	Q13424	Q61234	Q76EY8*	G1KHX2*	F7BBF2*	Q6GMG2*
α-dystrobrevin	Q9Y4J8	Q9D2N4	E1C4Z7*	H9GC72*	F7BTY8*	A2CJ03*

* Unreviewed protein identifier.

**Figure 3:**
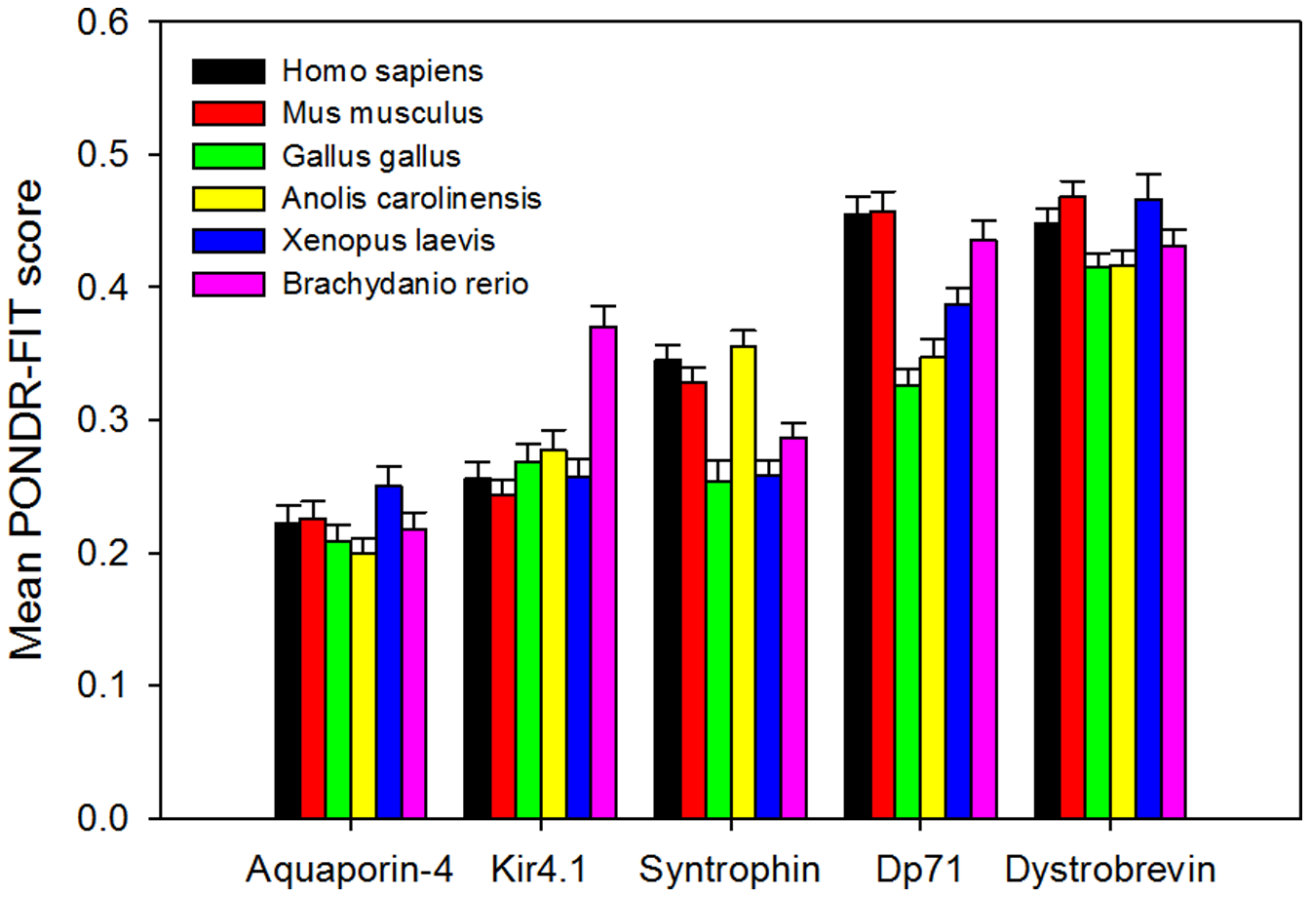
Mean disorder scores evaluated for five DAPC proteins from six vertebrate species: *Homo sapiens* (black bars), *Mus musculus* (red bars), *Gallus gallus* (green bars), *Anolis carolinensis* (yellow bars), *Xenopus laevis* (blue bars), and *Brachydanio rerio* (pink bars). Error bars represent the corresponding standard errors.

Looking at each plot globally, trends of order and disorder can still be seen. Here, the amount of predicted disorder among the various DAPC proteins increases in the following order: AQP-4<Kir4.1<α-1 syntrophin <Dp71<α-dystrobrevin. Figure 3 further illustrates this conclusion by showing mean disorder scores evaluated for all analyzed proteins. Another interesting observation is that for any given DAPC member, the overall appearance of the profiles of predicted disorder is rather conserved among various vertebrate species (see Figure 2). This finding suggests that both ordered and intrinsically disordered regions might play important roles in the functionality of the corresponding proteins.

### Structural Characterization of the DAPC Proteins

**Figure 4:**
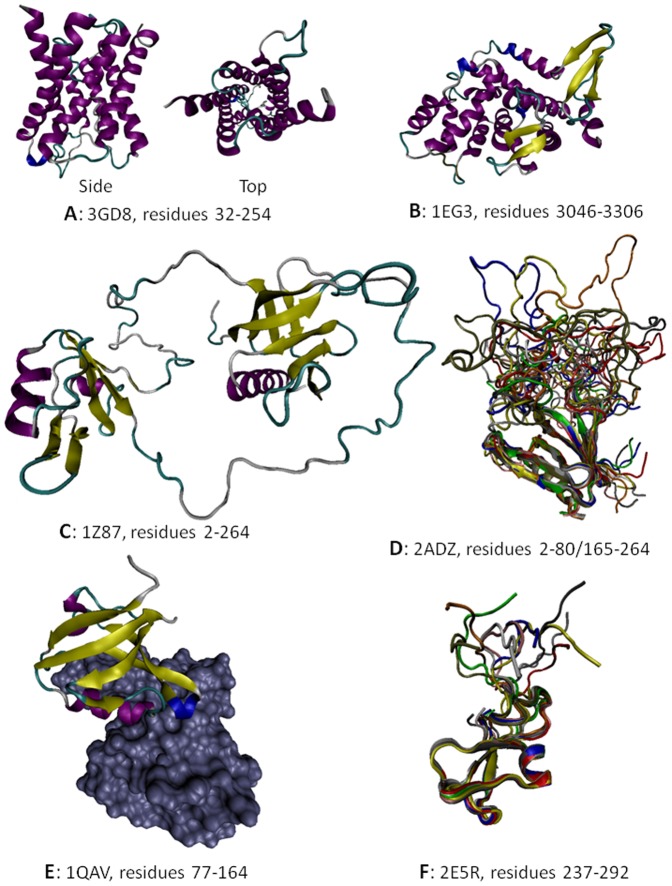
Structural information on some members of the dystrophin-associated protein complex: A. Crystal structure of the human AQP-4 fragment (PDB ID: 3GD8, residues 32–254); B. Crystal structure of the human dystrophin fragment (PDB ID: 1EG3, residues 3046–3306). **C**. NMR solution structure of the mouse α-1 syntrophin fragment (PDB ID: 1Z87, residues 2–264 that correspond to the PH_N_–PDZ–PH_C_ module). **D**. NMR solution structure of the mouse α-1 syntrophin fragment (PDB ID: 2ADZ, residues 2–80/165–264 that correspond to the PH_N_-‘L’-PH_C_ construct). Ten representative members of the conformational ensemble are shown by chains of different color. **E**. Crystal structure of a complex (PDB ID: 1QAV) between the PDZ domain of mouse α-1 syntrophin (residues 77–164, shown as a colored chain) and the Neuronal nitric oxide synthase, nNOS (shown as gray surface). **F**. NMR solution structure of the fragment of human α-dystrobrevin (PDB ID: 2E5R, residues 237–292 that correspond to the ZZ-domain). Ten representative members of the conformational ensemble are shown by chains of different color.

Figure 4 summarizes currently available information on the 3-D structures of proteins from the DAPC. Figure 4A represents side and top views of the structurally characterized domain of AQP-4 corresponding to the central 32–254 region, which is predicted to be mostly ordered (see Figure 2A). Despite the fact that the central region of Kir4.1 is expected to be mostly ordered (see Figure 2B), no structural information is available for this protein as of yet. Structure of a part of the C-terminally located fragment of dystrophin (residues 3046–3306) that roughly corresponds to the N-terminal half of Dp71 is shown in Figure 4B. Dp71 (622 residues) is the fifth isoforms of the human dystrophin produced by the alternative splicing. Dp71 differs from the canonical full-length protein (residues 1–3685) by lacking the first 3068 residues, missing the residues 3409–3421, having the residues KVPYYIN (3069–3075) changed to MREQLKG, and with the 3673–3685 region (residues RNTPGKPMREDTM) being changed and extended to HNVGSLFHMADDLGRAMESLVSVMTDEEGAE. The crystallized fragment of human dystrophin includes the WW-domain (residues 3055–3088) and is clearly predicted to be mostly ordered (see region corresponding to the first 300 residues in Figure 2C).

Although structural information on human α-1 syntrophin is not available as of yet, the solution structure of the N-terminal domain of its mouse counterpart has been determined (see Figure 4C). It is known that all five members (α, β1, β2, γ1, and γ2) of the syntrophin family share the same domain organization: an N-terminal split pleckstrin homology (PH) domain containing an embedded PDZ domain (PH_N_–PDZ–PH_C_), a central PH domain, and a C-terminal syntrophin unique domain (SU) [110]–[114]. In a mouth α-1 syntrophin split PH-domain, the PH_N_ half is composed of three β-strands (β1–β3), and the PH_C_ half contains the remaining four β-strands (β4–β7) and the C-terminal α-helix. A well-folded PDZ domain and two long linkers are inserted at the β3/β4-loop of the PH domain. Figure 4C shows a solution structure of one of the members of the conformational ensemble of the PH_N_–PDZ–PH_C_ module. Here, both PDZ and split PH-domains are well-folded and separated by highly flexible linkers [114]. This shows that PH_N_ and PH_C_ fragments fold together to form a canonical PH-domain structure containing seven β-strands and one C-terminal α-helix, and that PDZ-domian does not interfere with this folding process. Interestingly, the individual PH_N_ and PH_C_ fragments are completely disordered in isolation but fold into the canonical PH-domain, being mixed together [114]. When the PDZ-domain is taken out the PH_N_–PDZ–PH_C_ module and substituted by an eight-residue peptide linker (‘L’), the resultant PH_N_-‘L’-PH_C_ construct is able to fold into a structure indistinguishable from that of the joined PH_N_–PH_C_ domain within the PH_N_–PDZ–PH_C_ module (see Figure 4D) [114]. An interesting feature of this structure is the presence of a highly flexible β3/β4 loop. This structure provides further support to the notion that long flexible linkers are needed to ensure sufficient separation of the PDZ-domain from the halves of the split PH-domain thereby guarantying their ability to interact and mutually fold.

Finally, several crystal and NMR structures are available for the isolated PDZ-domain and for a complex of this domain with specific binding partners. Figure 4F shows one of these complexes were the PDZ-domain of the mouse α-1 syntrophin was co-crystallized with the neuronal nitric oxide synthase (nNOS) [115]. Importantly, these ordered α-1 syntrophin domains mostly match regions of predicted order in this protein whereas noticeable regions of predicted disorder corresponds to the flexible linkers connecting PH_N_ to PDZ and PDZ to PH_C_ (see Figure 2F).

As far as human α-dystrobrevin is concerned, this protein contains several functional domains, such as a region of interaction with the melanoma-associated antigen E1 (MAGEE1, residues 1–288) that also contains a zinc finger domain of ZZ-type (residues 237–284), a syntrophin-binding region (residues 400–450) and a potential coiled-coil region (residues 46–556). The high abundance of intrinsic disorder within the C-terminal half of α-dystrobrevin (Figure 2E) suggests that defining crystal structure of this protein could be a challenge. In agreement with this hypothesis, the structural information is only available for the zinc finger domain (residues 237–292, see Figure 4F). Interestingly, although this region is predicted to be disordered (see Figure 2E), it folds as a result of binding of two Zn^2+^ ions (Figure 4F), as typically the case for many other zinc-binding proteins and zinc-finger domains [21]–[23]. Both, MAGEE1-interacting region and syntrophin-binding region are predicted to be mostly ordered (see Figure 2E).

### Intrinsic Disorder and Sequence Conservation Analysis of the Intersubunit Binding Sites

Since DAPC is formed and stabilized by a number of intersubunit interactions, the corresponding binding sites were next found based on the previously published experimental studies (see Table 2). At the next step, the found intersubunit binding sites of the DAPC members were subjected to the sequence conservation analysis which revealed that these binding sites are well conserved in six vertebrate species, two mammals (*Homo sapiens* and *Mus musculus*), bird (*Gallus gallus*), reptile (*Anolis carolinensis*), amphibian (*Xenopus laevis*), and fish (*Brachydanio rerio*).

**Table 2:** Intersubunit binding regions found in the DAPC members.

No.	A: B	Binding Site of A	Binding Site of B	Reference
1	AQP-4: α-1 syntrophin	319–323	PDZ (87–170)	[90], [126], UniProt
2	Kir4.1: α-1 syntrophin	375–379	PDZ (87–170)	[90], UniProt
3	α-1 syntrophin: Dp71	SU (449–505)	‘Syntrophin Domain’ (362–412)	[118], [119], UniProt
4	α-1 syntrophin: α-dystrobrevin	‘Dystrobrevin Domain’ (408–426)	‘Dp71 Domain’ (378–450)	[121], [122], UniProt
5	α-dystrobrevin: Dp71	‘Syntrophin Domain’ (460–500)	‘Dystrobrevin Domain’ (420–460)	[107]

C-terminal sequences of AQP-4 and Kir4.1 each contains short highly conserved regions (the 319–323 fragment in human AQP-4 (residues VLSSV) and the residues RISNV at the position 377–381 in human Kir4.1. These sequences are responsible for the AQP-4 and Kir4.1 interaction with the α-1 syntrophin PDZ-domain. Figure 2 shows that the PDZ-domain binding sites of both channels are located within their disordered C-terminal tails.

**Figure 5:**
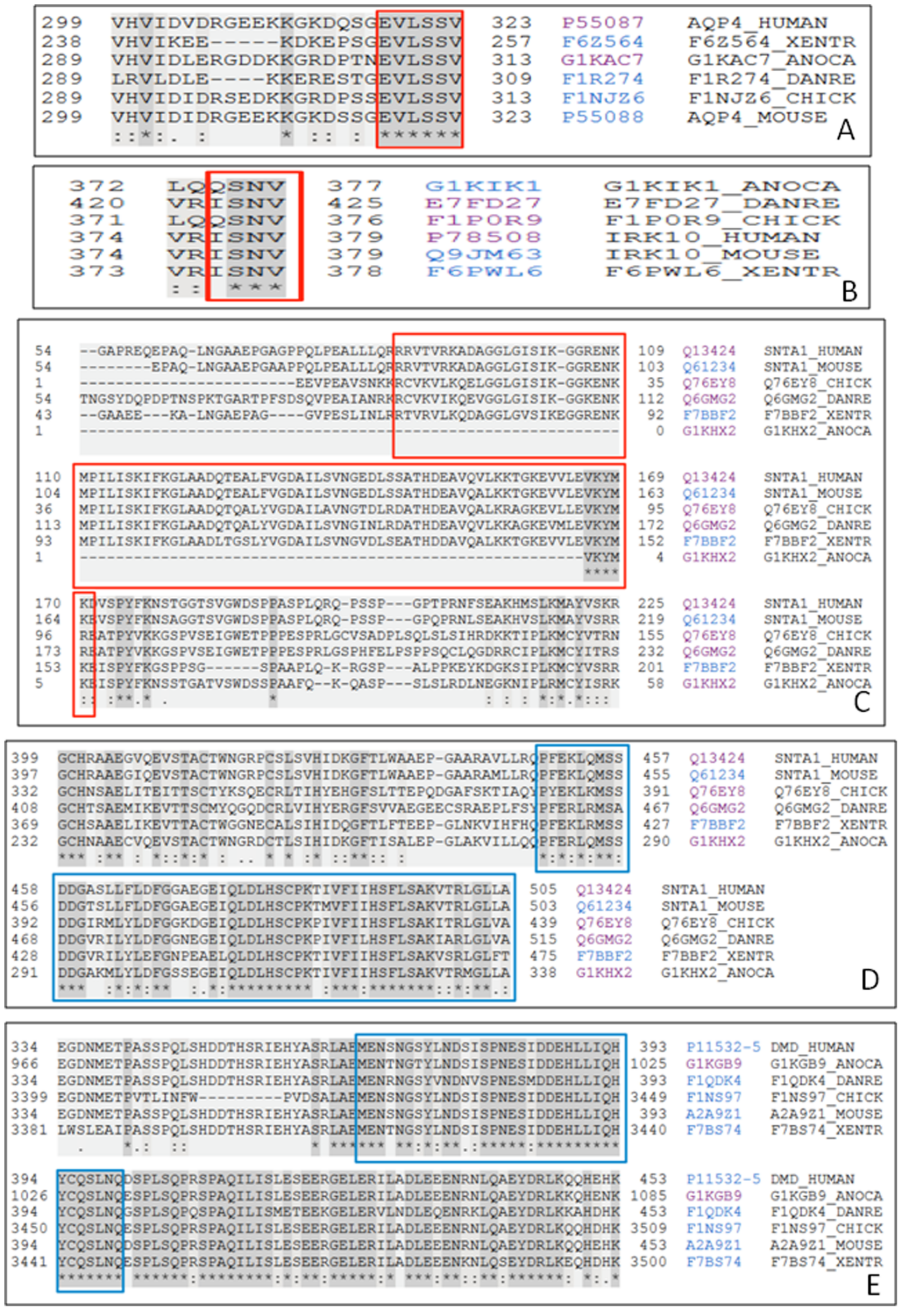
Sequence conservation of the PDZ-binding motifs in AQP-4 (A) and Kir4.1 (B), and of the α-1 syntrophin PDZ-domain (C). Sequence conservation of the α-1 syntrophin SU domain (**D**) and of the α-1 syntrophin binding domain of Dp71 (**E**). In each plot, the position of the corresponding binding motifs and functional domains are highlighted as a colored rectangle.

As it was mentioned above, human and mouse α-1 syntrophins have similar domain organization. The human protein consists of an N-terminal split PH domain (residues 6–86 and 171–268) containing an embedded PDZ domain (residues 87–170), a central PH domain (residues 293–401), and a C-terminal SU-domain (residues 449–505). The PDZ domains of α-1 syntrophin (residues 87–170 in human protein) are known to bind to the last three or four amino acids of ion channels and receptor proteins [116], [117]. Evolutionary analysis revealed that the PDZ-domain of α-1 syntrophin as well as the PDZ-binding motifs of AQP-4 and Kir4.1 are highly conserved among vertebrates (see Figure 5).

The SU-domain of α-1 syntrophin (residues 449–505 in human protein) is known to interact with specific segments of the Dp71 and α-dystrobrevin proteins [116], [118], [119]. This SU domainis mostly conserved among the vertebrates (Figure 5D). Significant portion of the SU-domain, including its calmodulin-binding subdomain (residues 481–503 in human protein) [120], is predicted to be disordered (see Figure 2F).

**Figure 6:**
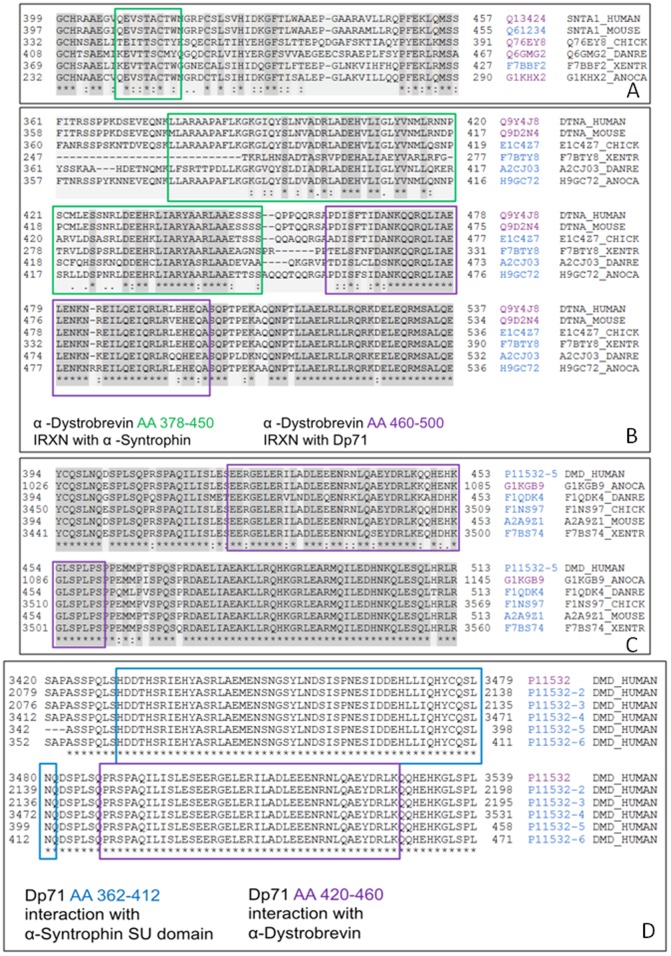
Analysis of sequence conservation of the inter-subunit interaction sites in the DAPC. In each plot, the position of the corresponding binding motifs and functional domains are highlighted as a colored rectangle. **A**. Conservation of the α-dystrobrevin-interacting site of α-1 syntrophin. **B**. Multi-domain sequence conservation analysis of α-dystrobrevin (conservation of the domains interacting with α-1 syntrophin and Dp71). **C**. Conservation of the α-dystrobrevin-interacting domain of Dp71. **D**. Multi-domain sequence conservation analysis of Dp71 (conservation of the domains interacting with the α-1 syntrophin SU-domain and α-dystrobrevin).

The portion of the molecule involved in binding to the α-1 syntrophin SU-domain spans amino acids 362–412. This interactive Dp71 segment, being mostly disordered (see Figure 2C), is highly conserved among vertebrates (Figure 5E). The interacting regions between α-1 syntrophin (residues 408–416) and α-dystrobrevin (residues 378–450) are also known [121], [122]. Figure 2 suggests that although the α-1 syntrophin-interacting domain of α-dystrobrevin is located within the predominantly ordered region, the α-dystrobrevin-binding motif of α-1 syntrophin is predicted to possess noticeable conformational flexibility (as indicated by its disorder scores noticeably deviating from 0.0). Curiously, conservation analysis revealed that the α-dystrobrevin-binding motif of α-1 syntrophin possesses minimal conservation among different species (Figure 6A), whereas the mostly disordered α-1 syntrophin-interacting domain of α-dystrobrevin showed more cross-species conservation (Figure 6B). Figure 2 shows that the interacting regions between the Dp71 (residues 420–460) and α-dystrobrevin (residues 460–500) are predicted to be highly disordered. Despite this fact, these intersubunit binding regions were shown to be highly conserved in the vertebrate species (see Figures 6B and 6C). Finally, since the Dp71 is one of the alternatively spliced isoforms of a canonical dystrophin, we analyzed the inter-isoform conservation of the dystrophin domains interacting with the SU-domain of α-1 syntrophin and with α-dystrobrevin and showed that these two binding domains are highly conserved among all the dystrophin DMD isoforms (Figure 6D).

Therefore, these analyses revealed that the intersubunit binding regions of the DAPC proteins are highly conserved. Importantly, our analysis also showed that in each interacting pair analyzed, at least one binding region involved in such intersubunit contacts was predicted to be intrinsically disordered. These results indicated that the DAPC proteins utilize highly conserved IDPRs for their intersubunit communications, thereby emphasizing the functional importance of intrinsic disorder in assembly of this important complex.

### Predicted Intrinsic Disorder-Based Binding Sites in the DAPC proteins

We also analyzed the coincidence of known binding sites of various DAPC proteins with the potential binding sites predicted by two principally different computational tools for finding the disorder-based interaction features, the ANCHOR (http://anchor.enzim.hu/) [123] and the MoRFpred (http://biomine-ws.ece.ualberta.ca/MoRFpred/ index.html) [124]. Results of this analysis are summarized in Table 3 which clearly shows that the majority of known binding sites are predicted by at least one of these computational tools. This finding provides further support to the idea that intrinsic disorder is important for the DAPC formation and stabilization, since many binding motifs of the DAPC proteins are located within the disordered regions. Also, it is obvious that there is some kind of disorder complementarity among binding sites, since when one interacting protein displays interaction-prone regions of intrinsic disorder, the binding site of its binding partner typically does not contain disorder. In this way, one protein acts as a donor, offering its intrinsically disordered region for binding, while the other protein acts as an acceptor, offering its more rigid region to complement the binding interaction.

**Table 3:** Comparison of the ANCHOR and MoRFpred results with known binding site.

No.	Protein	ANCHOR	MoRFpred	Already known
1	AQP-4	None	319–323	319–323
2	Kir4.1	None	375–379	375–379
3	α-1 syntrophin	113–119, 129–135, 163–170	None	87–170, 449–505, 408–426
4	Dp71	362–401, 409–412, 420–460	387–395, 423–430, 442–449	362–412, 420–460
5	α-dystrobrevin	379–396, 406–416, 437–445	378–384, 434–442, 466–475, 477–480	378–450, 460–500

### Intrinsic Disorder and Diseases-Causing Mutations in the DAPC proteins

**Figure 7:**
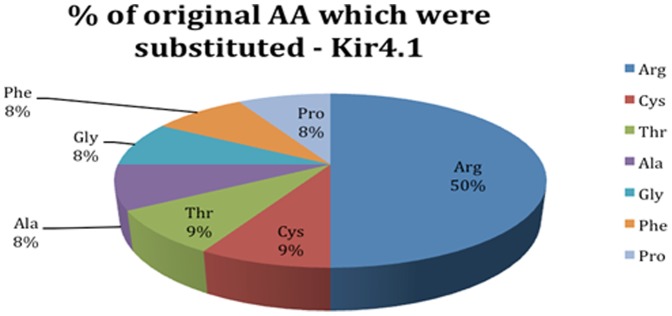
Frequencies of disease-related mutations in Kir4.1.

Next we analyzed the human DAPC proteins for the presence of mutations known to cause diseases. This analysis did not find reported mutations in the AQP-4 protein that were known to lead to disease phenotypes. However, twelve allelic variants were found in Kir4.1. Analysis of these mutations revealed that half of the allelic variants affected arginine (see Figure 7). The complete list of substitutions found in Kir4.1 and the positions at which they occurred are shown in Table 4. It is important to note that no disease-promoting mutations were found in the known binding site of Kir4.1 (amino acids 375–379).

**Table 4:** Disease-related amino acid substitutions in Kir4.1.

Original	Substitution	Position
Arg	Pro	65
Arg	Cys	65
Phe	Leu	75
Gly	Arg	77
Pro	Leu	121
Cys	Arg	140
Thr	Ile	164
Ala	Val	167
Arg	His	194
Arg	Ter	199
Arg	Cys	297
Arg	Cys	348

Eleven disease-related mutations were identified in the Dp71-encoding gene *DMD*. In most cases, these mutations result in Duchenne muscular dystrophy (see Table 5). Six out of the eleven mutations were frame shifts downstream from either a Lysine or Leucine residue, causing the protein to be truncated. In addition, point mutations, which caused arginine to change into a stop codon, were observed three times at positions 122, 302 and 313. Interestingly, there were three frame-shift mutations, in the α-1 syntrophin binding site of Dp71, at positions 363, 404, and 411. Mutations of two of these positions (Leu363 and Leu404) lead to the frame shifts resulting in the Duchenne muscular dystrophy, whereas the mutation of Ala411 was another frame-shift mutation that caused Becker muscular dystrophy.

**Table 5:** Disease-related amino acid substitutions in Dp71.

Original	Effect	AA Position
Ile	Frame shift	89
Arg	Termination	122
Arg	Termination	122
Cys	Substitution to Tyr	272
Arg	Termination	302
Lys	Frame shift	306
Arg	Termination	313
Leu	Frame shift	363
Leu	Frame shift	404
Ala	Frame shift	411
Leu	Frame shift	414

The only disease-related mutation in the α-1 syntrophin is an A390V mutation which is linked to long QT syndrome 12. However, this mutation is positioned outside the PDZ- and SU-domains as well as outside the region that interacts with α-dystrobrevin. Finally, only one allelic variant (P121L) is known for α-dystrobrevin, which is also located outside any relevant binding sites.

Because, intrinsic disorder is an important function-related feature, we analyzed whether the known SNPs can cause significant changes in the intrinsic disorder pattern of whole protein. To this end, a paired T-test between the wild type protein and each variant containing a single SNP was performed. Here, we analyzed the statistical significance of the single SNP effect on the disorder probabilities of proteins evaluated by PONDR-FIT, PONDR® VLXT, and PONDR® VSL2. Here, a paired T-test was applied to a pair of averaged per residue disorder scores calculated for the wild-type protein and variant and significance level of 0.05 was used to determine the statistical significance of the effect of a given SNP. Among numerous SNPs reported for each protein, only SNPs affecting binding sites and disease causing SNPs were considered. The analysis of SNPs affecting residues in the binding sites or binding domains revealed that the majority of such mutations caused the related protein to be less disordered than the corresponding wild type protein (i.e., the majority of SNPs resulted in some decrease in the overall disorder score of a corresponding protein) (Table 6). Here, among 40, 39 and 39 SNPs analyzed by PONDR-FIT, PONDR® VLXT, and PONDR® VSL2, there were 24, 29, and 25 SNPs, respectively, which were predicted to make whole protein to be less disordered. Similarly, the majority of disease-causing SNPs tend to change each protein to be less disordered than the corresponding wild type protein (see Table 7). Again, among 15-15-15 SNPs checked by PONDR-FIT, PONDR® VLXT, and PONDR® VSL2 algorithms, 7, 13 and 6 SNPs were predicted to decrease the mean disorder score of a corresponding protein. Importantly, there was no intersection between the SNPs in binding sites and disease causing SNPs. To further substantiate our conclusions, we also analyzed the effect of disease-causing SNPs located within the functional motifs and functional domains that are listed for the DACP members in the ELM (http://elm.eu.org/) and pFam (http://pfam.sanger.ac.uk/) databases. The corresponding data are listed in Table 8.

**Table 6:** Paired T-test P-values of the SNPs in the intersubunit binding sites.

Protein	SNP	PONDR-FIT (effect)	PONDR-VLXT (effect)	VSL2 (effect)
AQP-4	V319I	1.72e-2 (Less ID)	1.62 e-3 (Less ID)	1.51e-9 (Less ID)
Kir4.1	R375H	7.88e-7 (Less ID)	1.26e-3 (Less ID)	1.90e-8 (Less ID)
Dp71	N399K	8.74e-9 (Less ID)	1.47e-6 (Less ID)	9.21e-8 (Less ID)
	Q400R	7.57e-11 (Less ID)	4.76e-6 (Less ID)	2.18e-11 (Less ID)
	I428V	1.99e-7 (Less ID)	4.98e-7 (More ID)	2.84e-11 (Less IDP)
	R445H	1.65e-6 (Less ID)	3.98e-6 (Less ID)	4.41e-12 (Less ID)
	R445L	2.81e-10 (Less ID)	4.32e-6 (Less ID)	3.05e-13 (Less ID)
	R445P	4.17e-4 (Less ID)	None	None
	S456T	1.08e-7 (Less ID)	1.88e-5 (Less ID)	1.51e-14 (Less ID)
	S456Y	6.17e-12 (Less ID)	6.18e-5 (Less ID)	1.98e-15 (Less ID)
	L458V	3.31e-12 (Less ID)	3.33e-5 (More ID)	1.31e-15 (Less ID)
α-1 syntrophin	R106Q	4.75e-4 (Less ID)	5.68e-6 (Less ID)	4.90e-1 (Less ID)
	I114F	1.13e-7 (Less ID)	2.05e-5 (Less ID)	8.81e-9 (Less ID)
	K116E	1.24e-8 (More ID)	8.99e-6 (More ID)	1.63e-4 (Less ID)
	A122T	3.63e-3 (Less ID)	4.92e-6 (Less ID)	7.71e-4 (More ID)
	Q125H	1.61e-1 (Not significant)	5.44e-5 (Less IDP)	1.07e-1 (Not significant)
	F130L	1.11e-1 (Not significant)	2.21e-6 (More IDP)	5.04e-7 (More ID)
	S144A	6.13e-8 (Less ID)	4.87e-7 (Less ID)	2.53e-8 (Less ID)
	T147N	1.32e-4 (More ID)	5.62e-7 (Less ID)	3.32e-3 (More ID)
	V154I	4.64e-6 (Less ID)	2.21e-5 (Less ID)	6.81e-11 (Less ID)
	R419C	3.82e-6 (Less ID)	1.87e-4 (Less ID)	7.86e-6 (Less ID)
	E451K	2.43e-3 (Less ID)	2.14e-6 (Less ID)	1.68e-3 (More ID)
	G460S	2.96e-6 (More ID)	5.08e-7 (More ID)	1.42e-6 (More ID)
	L463R	2.48e-4 (More ID)	9.35e-5 (Less ID)	1.17e-5 (More ID)
	S481L	1.36e-2 (More ID)	5.70e-5 (Less ID)	1.07e-4 (Less ID)
	S495-	1.13e-4 (More ID)	2.50e-1 (Not significant)	1.31e-2 (More ID)
	S495L	3.93e-1 (Not significant)	1.95e-3 (Less ID)	1.34e-4 (Less ID)
α-dystrobrevin	A383S	6.72e-10 (More ID)	3.82e-5 (More ID)	3.36e-9 (More ID)
	N397D	1.02e-5 (More ID)	1.95e-1 (Not significant)	1.59e-8 (More ID)
	A403V	9.21e-12 (Less ID)	8.18e-7 (Less ID)	6.58e-9 (Less ID)
	H406Y	2.33e-7 (Less ID)	3.53e-5 (Less ID)	9.25e-10 (Less ID)
	G410R	1.33e-9 (More ID)	2.68e-5 (Less ID)	4.59e-10 (More ID)
	Y412S	1.28e-13 (More ID)	1.76e-7 (More ID)	6.58e-12 (More ID)
	R417W	6.83e-15 (Less ID)	2.80e-6 (Less ID)	2.31e-9 (Less ID)
	C422R	3.79e-13 (More ID)	1.95e-2 (More ID)	2.55e-9 (More ID)
	E425G	4.21e-10 (Less ID)	1.03e-5 (Less ID)	1.63e-9 (Less ID)
	A445T	1.07e-4 (More ID)	2.84e-6 (Less ID)	1.93e-10 (Less ID)
	S448F	1.62e-13 (Less ID)	6.80e-7 (Less ID)	5.48e-11 (Less ID)
	D467N	1.97e-1 (Not significant)	5.96e-4 (Less ID)	9.20e-7 (More ID)
	R494W	6.21e-11 (Less ID)	4.17e-7 (Less ID)	3.68e-13 (Less ID)

**Table 7:** Paired T-test P-values of the disease-causing SNPs.

Protein	SNP	PONDR-FIT (effect)	PONDR-VLXT (effect)	VSL2 (effect)
AQP-4	None			
Kir4.1	R65P	8.64e-8 (More ID)	6.45e-4 (Less ID)	1.44e-3 (More ID)
	G77R	1.11e-8 (Less ID)	7.97e-5 (More ID)	2.83e-1 (Not significant)
	C140R	1.77e-1 (Not significant)	3.66e-7 (More ID)	2.18e-4 (More ID)
	T164I	2.30e-4 (More ID)	1.34e-4 (Less ID)	5.96e-4 (Less ID)
	A167V	4.41e-3 (Less ID)	4.89e-5 (Less ID)	2.14e-4 (Less ID)
	P194H	1.95e-1 (Not significant)	4.65e-5 (Less ID)	3.82e-3 (More ID)
	R199-	1.07e-3 (More ID)	2.85e-3 (Less ID)	1.54e-1 (Not significant)
	R297C	3.20e-8 (Less ID)	3.23e-6 (Less ID)	8.30e-5 (Less ID)
	R348C	5.50e-7 (Less ID)	4.41e-7 (Less ID)	9.84e-10 (Less ID)
Dp71	R122-	2.83e-4 (Less ID)	5.25e-5 (Less ID)	2.33e-1 (Not significant)
	C272Y	2.08e-7 (More ID)	1.76e-4 (Less ID)	3.29e-5 (More ID)
	R302-	1.78e-3 (More ID)	5.16e-3 (Less ID)	3.07e-1 (Not significant)
	R313-	5.06e-3 (More ID)	3.55e-2 (Less ID)	3.33e-1 (Not significant)
α-1 syntrophin	A390V	2.15e-4 (Less ID)	2.68e-7 (Less ID)	6.17e-7 (Less ID)
α-dystrobrevin	P121L	3.55e-3 (Less ID)	2.80e-4 (Less ID)	5.71e-4 (Less ID)

**Table 8:** Functional motifs and functional domains of the DAPC members affected by the disease causing SNPs.

Protein	SNP	pFam	ELM	P-value	IDPR	Tendency	Disease
Kir4.1	R65P	IRK	TRG_LysEnd_APsAcLL_1 LIG_PP1	8.64e-8	×	More ID	Seizure
	G77R		MOD_GSK3_1TRG_PEX_2	1.11e-11	×	Less ID	Seizure
	C140R		None	1.77e-1	×	Not significant	Seizure
	T164I		None	2.30e-4	×	More ID	Seizure
	A167V		None	4.41e-3	×	Less ID	Seizure
	P194H		None	1.95e-1	×	Not significant	Deafness
	R199-		None	1.07e-3	×	More ID	Seizure
	R297C		None	3.20e-8	×	Less ID	Seizure
	R348C		MOD_PKA_2 MOD_PLK	5.50e-7	×	Less ID	Deafness
Dp71	R122-	EF-Hand 2	LIG_FHA_1 LIG_MAPK_1	2.83e-4	×	Less ID	Muscle Dystrophy
	C272Y	ZZ	None	2.08e-7	×	More ID	Muscle Dystrophy
	R302-	pFam-B _2847	None	1.78e-3	×	More ID	Muscle Dystrophy
	R313-	pFam-B _2847	CLV_PCSK_FUR_1	5.06e-3	×	More ID	Muscle Dystrophy
α-1 syntrophin	A390V	PH, pFam-B_796 & _18143	None	2.15e-4	×	Less ID	Long QT Syndrome 12
α-dystrobrevin	P121L	EF-Hand 2 SporeIII_AE	None	3.55e-3	×	Less ID	Left Ventricular Noncompaction 1

Our analysis revealed that although all five proteins contain multiple SNPs, disorder-based binding regions of these DAPC members are rarely affected by mutations. This observation suggests that the functional versatility of IDPRs in AQP-4, Kir4.1, Dp71, α-1 syntrophin, and α-dystrobrevin precludes these regions from being mutated, hence showing least number of mutations in them. However, it is also likely that mutations of individual residues within the functional IDPRs of these proteins are well tolerated, since the evolutionary pressure may have shifted to maintaining global biophysical properties and structural malleability of the IDPRs to safeguard the critical protein functions [125].

### Visual Analyses of the Effects of SNPs on Protein Intrinsic Disorder Propensity

Going beyond the disease related mutations, further analysis was performed by looking at all available SNPs for each of the five proteins from the dystrophin-associated protein complex. As stated above, the Ensemble Genome Browser (http://useast.ensemble.org) was used to search for all SNPs related to the proteins in question. The total variation analysis of each gene showed that there were 2,775 SNPs for genes encoding for AQP-4, 730 SNPs for KCNJ-10 (Kir4.1), and 832, 2,563, and 2,427 SNPs for α-1 syntrophin, Dp71, and α-dystrobrevin, respectively. Only SNPs that corresponded to the actual amino acid substitutions in corresponding proteins were analyzed. Thus, any nonsense, non-coding, splice region, synonymous, 3′ or 5′ UTR variants, or any other type of variant that did not pertain to actual amino acid changes were omitted as this data related to areas of each protein transcriptome that would not impact the amino acid sequences of the resulting protein forms. There were 242, 67, 81, 69, and 115 amino acid substitutions-inducing SNPs in AQP-4, Kir4.1, α-1 syntrophin, Dp71, and α-dystrobrevin, respectively.

**Figure 8:**
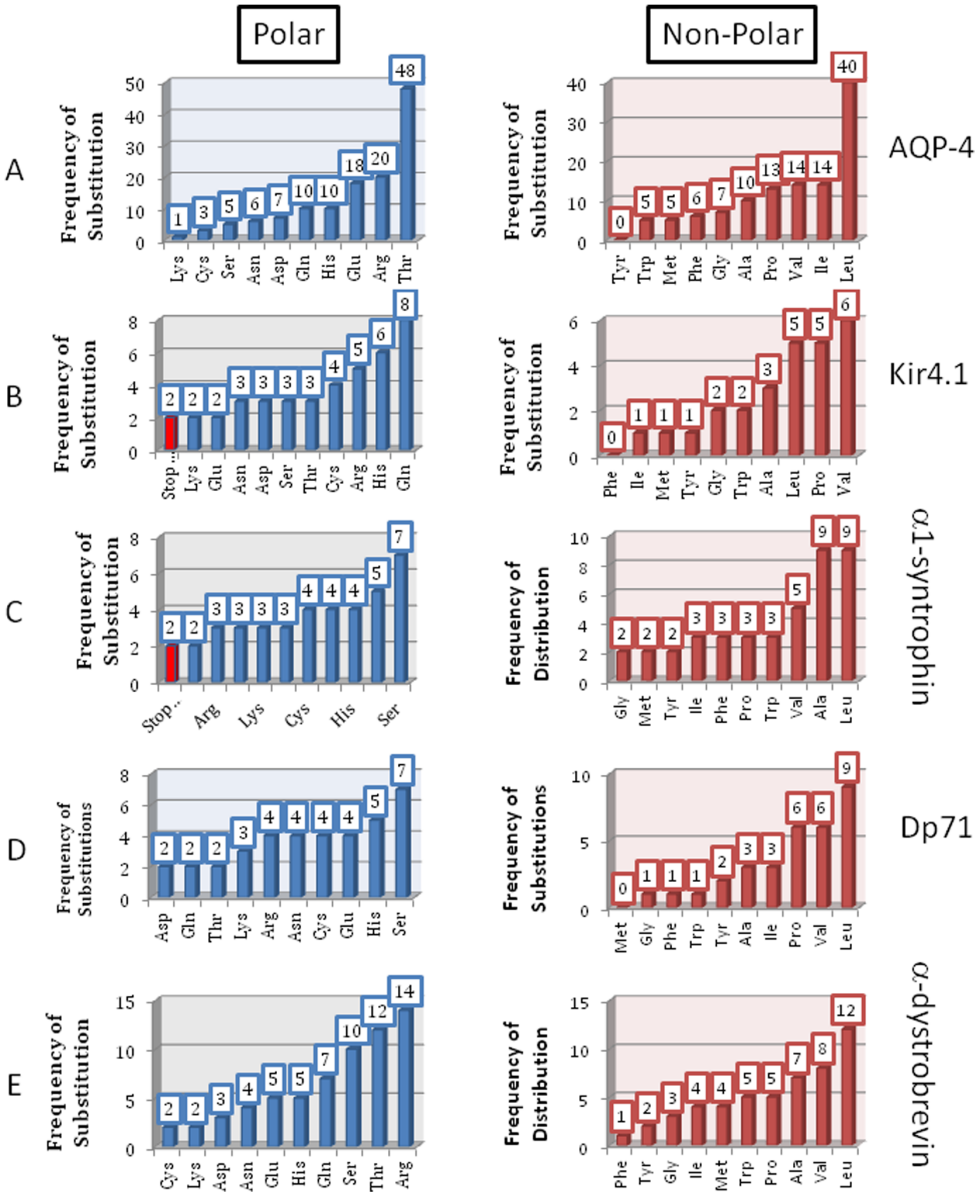
Distribution of SNPs affecting polar and non-polar residues in AQP-4 (A), KCNJ-10 (Kir4.1, B), α1-syntrophin (C), Dp71 (D), and α-dystrobrevin (E).

The analyzed variations were broken down into an overall distribution of substitutions affecting polar and non-polar residues in each protein. Figure 8 shows which amino acids dominate the total polar and non-polar substitutions for each protein and gives insights into the likelihood of SNPs and related disorders being linked to specific amino acids, as some are much more common than others. These distributions also help to conceptualize the potential effects of substitutions and make a prediction on whether a given mutation can affect protein folding and functionality. For example, the addition of a polar or charged residue to a protein that is mostly non-polar and hydrophobic (such as AQP-4 or Kir4.1) could have drastic impacts on the ability of this protein to maintain both stability and function and therefore to interact with the conjugate proteins in the complex. It is this exact occurrence that is the focus of our study.

**Figure 9:**
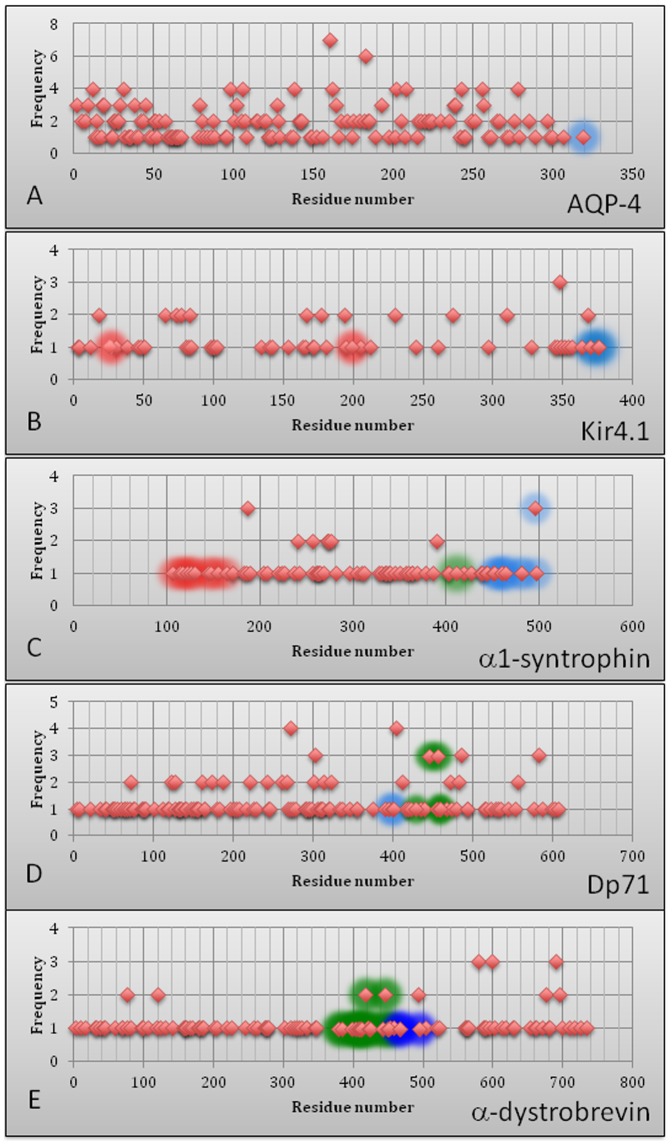
Scatter plots showing SNP distributions within the amino acid sequences of AQP-4 (A), Kir4.1 (B), α1-syntrophin (C), Dp71 (D), and α-dystrobrevin (E). SNPs happening in known binding regions are indicated by different colors that match colors in corresponding tables (see Tables 9-11).

Figure 9 represents a series of scatter diagrams that show the frequencies of substitutions within the corresponding protein sequences. Here, a high frequency number means that a certain position within the protein has a high level of SNP occurrence. These diagrams give an idea of where in the sequence certain variations are prone to happen. One should keep in mind though that multiple variations at a certain point in a protein can generate different residues. Areas of absence or abundance of points in these diagrams is a nice clue that can be linked to some other sequence specific features, such as distribution of intrinsic disorder propensities, thereby giving more insights in context of the overall stability/structure of the proteins of interest.

Specific SNPs are highlighted in each of these scatter plots to denote those that were present in specific binding regions. Different colors were used in order to distinguish between multiple binding regions present on a protein. All the different SNPs in binding regions of these proteins are given in Tables 9–11 and are color coded to indicate the regions in which they occur. Note, that a plot for the Kir4.1 protein has two colors to highlight four specific SNP's. While two SNPs (blue highlights, positions 375 and 376, Figure 9B) are associated with the actual binding region of this protein, the other two (red highlights, positions 26 and 199, Figure 9B) are SNPs resulting in gaining the stop-codons that lead to protein truncations. Therefore, these SNPs were considered important and included in the scatter plot for Kir4.1. The latter three scatter plots have many more SNPs associated with binding regions. However, as stated earlier no SNP within the binding regions of any of these proteins had known links to epileptic seizure phenotypes. Only Long QT Syndrome and multiple phenotypes of muscular dystrophy have been linked to mutations/variants in these binding regions.

**Table 9:** Binding site SNPs in α-1 syntrophin.

Region	SNP ID	SNP Type	Variation	AA Position
PDZ Domain (87–170, red)	rs75025585	Missense	R/Q	106
	TMP_ESP_20_32026803	Missense	I/F	114
	TMP_ESP_20_32026797	Missense	K/E	116
	rs151113230	Missense	A/T	122
	rs201421292	Missense	Q/H	125
	rs199964677	Missense	F/L	130
	rs142978180	Missense	S/A	144
	rs141724500	Missense	T/N	147
	rs200241523	Missense	V/I	154
	rs200647905	Synonymous	L	164
Dystrobrevin Domain (408–416, green)	rs142580715	Synonymous	V	410
SU Domain (449–505, blue)	rs116747979	Synonymous	F	450
	rs148604302	Missense	E/K	451
	rs146134721	Synonymous	D	459
	TMP_ESP_20_31996554	Missense	G/S	460
	rs188835994	Missense	L/R	463
	rs201485963	Synonymous	H	480
	TMP_ESP_20_31996389	Missense	S/L	481
	TMP_ESP_20_31996346	Synonymous	S	495
	rs144006909	Missense	S/L	495
	rs144006909	Stop gained	S/*	495
	rs34901081	Synonymous	A	496

**Table 10:** Binding site SNP in Dp71.

Region	SNP ID	SNP Type	Variation	AA Position
Syntrophin Domain (362–412, blue)	rs190867451	Synonymous	C	395
	TMP_ESP_X_31187673	Missense	N/K	399
	TMP_ESP_X_31187671	Missense	Q/R	400
Dystrobrevin Domain (420–460) green	rs149723217	Missense	I/V	428
	rs139547504	Missense	R/H	445
	COSM216862	Missense	R/L	445
	COSM216863	Missense	R/H	445
	rs150957972	Missense	S/T	456
	rs145123497	Missense	S/Y	456
	TMP_ESP_X_31165574	Missense	L/V	458
	rs72466538	Synonymous	P	459

**Table 11:** Binding site SNPs in α-dystrobrevin.

Region	SNP ID	SNP Type	Variation	AA Position
Syntrophin Domain (378–450, green)	rs192673085	Synonymous	L	378
	rs141881401	Missense	A/S	383
	rs150679265	Missense	N/D	397
	TMP_ESP_18_32418744	Missense	A/V	403
	rs139872140	Missense	H/Y	406
	TMP_ESP_18_32418763	Synonymous	I	409
	TMP_ESP_18_32418764	Missense	G/R	410
	rs186573363	Missense	Y/S	412
	rs199867593	Missense	R/W	417
	COS122724	Missense	R/W	417
	TMP_ESP_18_32418800	Missense	C/R	422
	TMP_ESP_18_32428268	Missense	E/G	425
	rs144776465	Synonymous	A	441
	COSM221429	Missense	A/T	445
	COSM221430	Missense	A/T	445
	rs147541731	Missense	S/F	448
	rs202088347	Synonymous	S	450
Dp-71 Domain (460–500, blue)	rs145061501	Synonymous	I	466
	rs144880521	Missense	D/N	467
	rs149071180	Synonymous	L	495

The results above show that only one SNP (rs200498749) was present in the AQP-4 binding region, at position 319. This was a missense variant that caused the amino acid change from valine to isoleucine. This mutation is located at the C-terminal end of the protein which is responsible for interacting with α-1 syntrophin. As stated earlier, this SNP is not known to be associated with any disease. The same was true for our analysis of Kir4.1 which showed an SNP at position 376, which is in the C-terminally located binding region of this protein. However, as with AQP-4, this SNP is not associated with any disease phenotypes. These two proteins are thought to be directly responsible for certain seizure phenotypes because of the role they play in the DAPC and cell homeostasis. However, the scatter plots show that SNPs are frequent outside of their short binding regions. This could mean that although the biding regions are primarily responsible for these proteins ability to interact, they have little to do with sustaining stability and overall protein functionality.

The scatter plots for α-1 syntrophin, Dp71 and α-dystrobrevin show a very different variation content as compared to those of the Kir4.1 and AQP-4 proteins. These plots emphasize the large difference in SNP frequency, especially in the binding regions of interest. As Figure 9 shows, the large majority of variations only have a frequency of one. This frequency forms the linear clustering that can be seen across the bottom of each respective scatter plot. Tables 9–11 give even more detail on these proteins, listing the ID, type, actual variation and amino acid position of each SNP as it relates to the particular binding site. It is important to note that a single amino acid position can be affected by multiple SNP types. This is best represented in the SU-domain of α-1 syntrophin where position 495 can have three different variations; a missense variant, a synonymous variant and a stop-codon variant. Although each of these SNPs can occur at the same location, they have drastically different impacts on the protein. The synonymous variant does not induce amino acid change, while the variant resulting in the stop-codon cuts off the last 11 amino acids, all of which are part of the SU-domain. As this domain is the region responsible for α-1 syntrophin interaction with both Dp71 and α-dystrobrevin, this SNP could have a significant impact on the DAPC and its overall stability/function. Therefore, the scatter plots shown in Figure 9 and the associated data help to uncover meaningful results from the SNP data as well as aid in the visualization of where these variations are physically occurring within each proteins amino acid sequence.

Further analysis was performed on the available SNPs for each of these five proteins in order to determine the impact that specific variations had on protein intrinsic disorder propensity that can be translated to the potential effects of amino acid variations on protein stability and functionality. At this stage we got rid of any identical SNPs which were repeated multiple times at a specific location. However, those SNPs which occurred at the same location but resulted in different amino acids were included. This allowed us to trim down the total variants of each protein to just those that were unique and which directly impacted the amino acid sequences. This pre-filtering generated 52, 56, 57, 56, and 80 unique SNPs for AQP-4, KCNJ-10 (Kir4.1), α-1 syntrophin, Dp71, and α-dystrobrevin, respectively that were used in all subsequent analyses.

**Figure 10:**
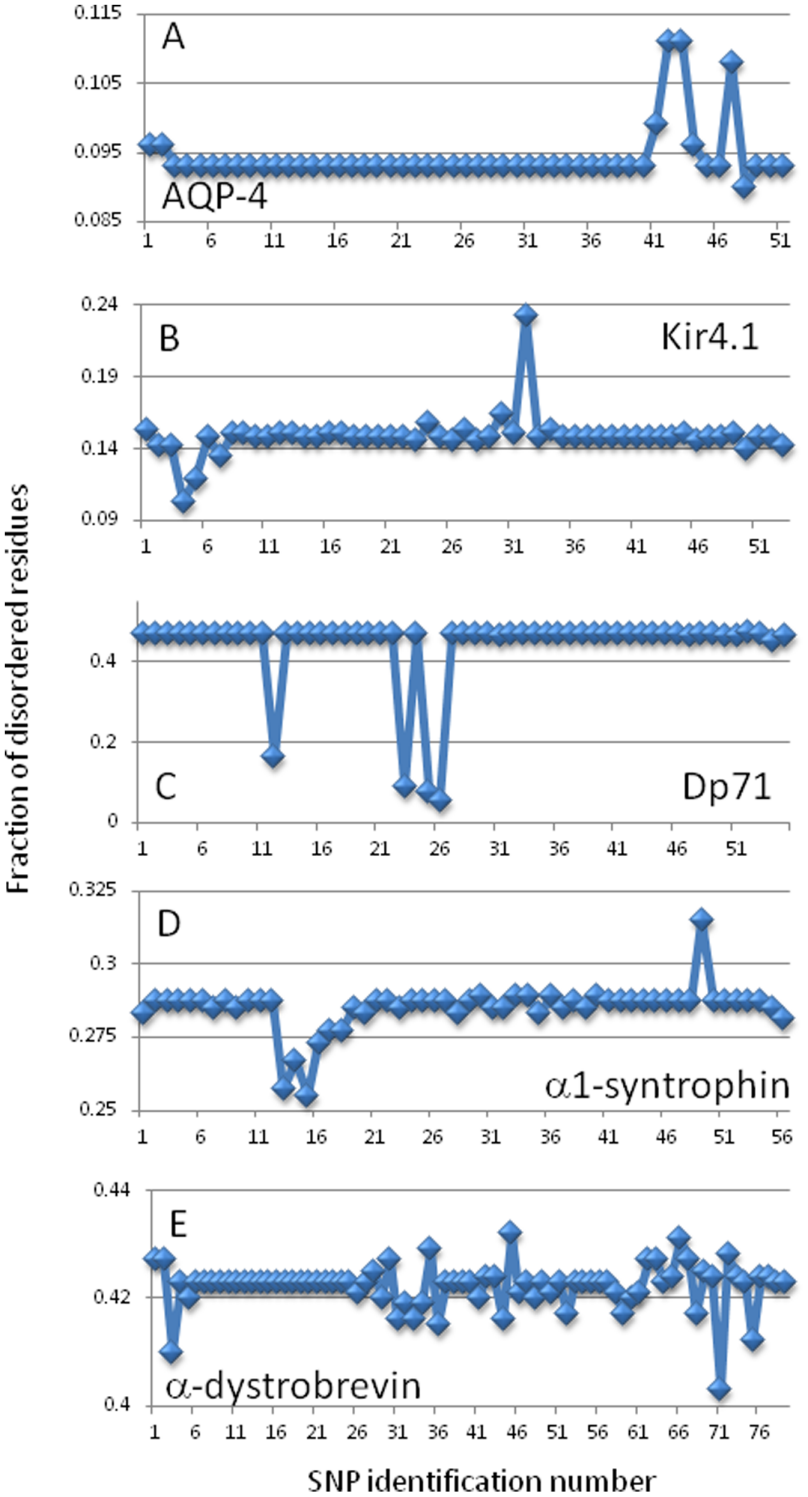
Analyzing the effect of SNPs on the fraction of disordered residues in a target protein: AQP-4 (A), Kir4.1 (B), Dp71 (C), α1-syntrophin (D), and α-dystrobrevin (E). For a given protein, faction of disordered residues was determined as a relative content of residues with the disorder score above the 0.5 threshold. Note that numbers on the X-axis correspond to the identification numbers of SNPs and not to their positions within the protein sequence.

Figure 10 is a visual representation of data on the effect of SNPs on the mean intrinsic disorder score of a given protein. The statistical significance of the effects of corresponding mutations on protein's disorder score was already discussed above. In each plot of the Figure 10, disorder scores are shown on the Y-axis, whereas X-axis represents the SNP numbers. Note that these SNP numbers are used as identifiers of given SNPs and are not related to the SNP positions within the protein sequence. Figure 10 shows that some proteins have very little variation associated with their SNPs while others have a wide range of differences. Additionally, proteins differ from each other not only by the number of SNPs affecting their mean disorder scores, but also by the amplitudes of these changes. Therefore, these plots provide a simple illustrative mean to discern which proteins are more likely to be effected by changes in their amino acid sequences. For example, the comparison of plots corresponding to AQP-4 and α-dystrobrevin shows that AQP-4 has only a few spots of extreme deviation from the wild type values, whereas α-dystrobrevin has numerous spots that differ from the wild type disorder value. This suggests that the disorder propensity of AQP-4 is much less affected by most variations. We believe that these types of graphs are helpful in that they give a clear picture of those SNPs which do or do not affect the disorder status of a protein. In this way, the number of unique SNPs which have to be focused on for further analysis can be trimmed down. Also, SNPs that do affect the mean disorder level can be ranked based on the severity of their effects (the scale of deviation from the mean disorder scores of wild type proteins), which provides another way to focus on specific variations.

**Figure 11:**
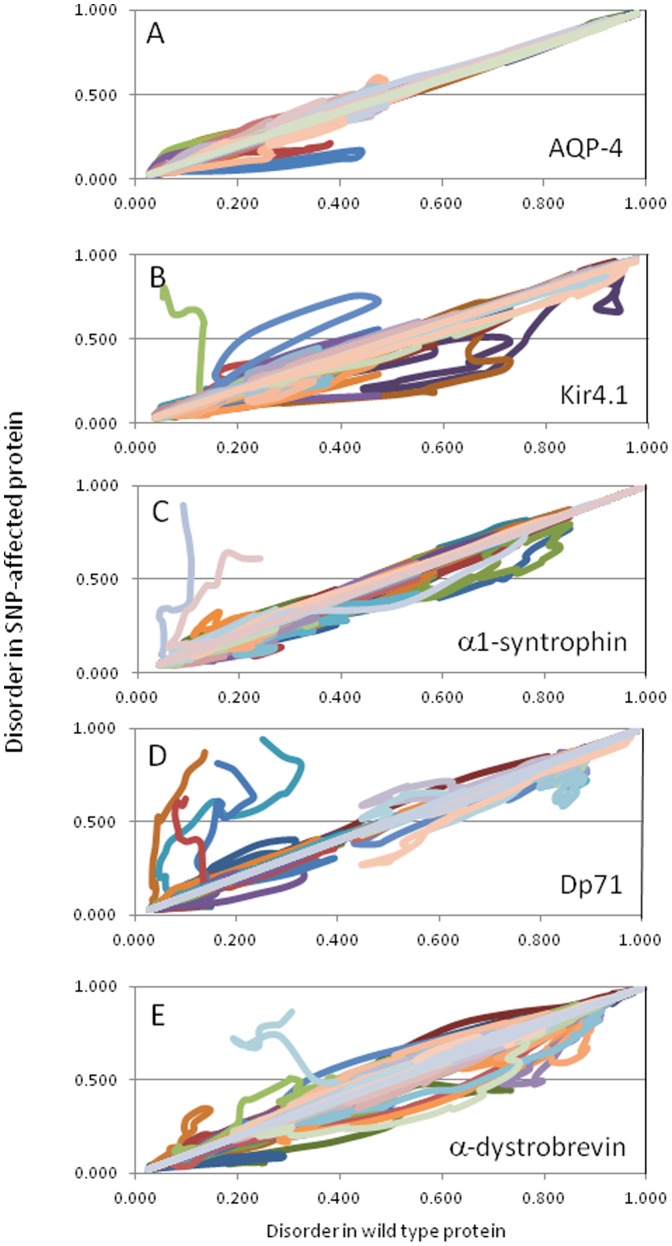
Correlation between the per-residue disorder scores of the wild type proteins and SNP-produced variants of AQP-4 (A), Kir4.1 (B), α1-syntrophin (C), Dp71 (D), and α-dystrobrevin (E). These graphs are generated by plotting the per-residue disorder scores of SNP-produced variants versus the per-residue disorder scores of corresponding wild type proteins. Each line in these plots corresponds to the pre-residue disorder scores correlation evaluated for one SNP-produced variant.

Another way of showing the effects of SNPs on the protein's disorder propensity is a plot where the per-residue disorder scores of the SNP-produced variant are correlated with the per-residue disorder scores of the corresponding wild type protein (see Figure 11). In corresponding plots, the X-axis shows the per-residue disorder scores for the wild type proteins, whereas the Y-axis shows the position matched disorder scores of the mutant protein. Obviously, when mutations do not affect the protein's disorder score, the corresponding dependence is described as a straight line following the diagonal of a given plot, whereas any deviation from this diagonal straight line is a representation of an effect of an SNP on protein's disorder propensity. Here, the positive deviations (i.e., moving plots above the diagonal) reflect the SNP-induced increase of intrinsic disorder propensity in a given protein, whereas the negative deviations (i.e., those moving plots below the diagonal) denote the SNP-promoted decrease in protein's disorder level. Obviously, the severity of the effect of an SNP on protein disorder propensity can be evaluated by the magnitude of the corresponding line deviation from the diagonal.

Figure 11A represents the corresponding disorder correlation graph for AQP-4 that has only a few SNPs that significantly shift the disorder line away from the diagonal. The three that stand out the most are SNPs at positions 136 (Valine-Phenylalanine), 182 (Arginine-Tryptophan), and 260 (Arginine-Cysteine). Figure 11A shows that these three variants pushed the protein towards more order. Most changes in disorder propensity happen in a window of about 15–30 amino acids surrounding the SNP. The range seems to be dependent on how different the amino acid is from the wild type and the characteristics of the residues surrounding it.

Figure 11B shows that Kir4.1 has five SNPs causing severe shifts in protein's order/disorder propensity. These are SNPs at positions 18 (Arginine-Tryptophan), 26 (Arginine-Stop Codon), 36 (Arginine-Cytosine), 171 (Arginine-Glutamine), and 181 (Phenylalanine-Leucine). The stop codon at position was the most severe as it truncated the protein to a length of only 25 residues and caused noticeable increase in protein's disorder propensity. The SNP at position 181 also increased disorder noticeably. The other three SNPs, 18, 36 and 171, all resulted in increased order in a protein.

α-1-Syntrophin was mostly unaffected by a majority of SNP's. However, Figure 11C shows that there is a general shift below the diagonal which would denote more order in variants produced by SNPs. The SNPs at positions 189 (Serine-Leucine), 207 (Arginine-Tryptophan), and 495 (Serine-Leucine) were the most order-promoting, whereas the SNPs affecting residues 442 (Arginine-Stop Codon) and 495 (Serine-Stop Codon) both truncated the protein and caused significant increases in disorder.

Figure 11D illustrates that the SNP-induced variability of the Dp71 disorder propensity is rather high. Four SNPs, 122 (Arginine-Stop Codon), 242 (Glutamine-Stop Codon), 302 (Arginine-Stop Codon) and 313 (Arginine-Stop Codon), incorporated stop codons and resulted in the great increase in the disorder propensity. SNPs that occur at positions 7 (Glycine-Serine), 64 (Alanine-Aspartic Acid), 583 (Asparagine-Lysine), and 583 (Asparagine-Serine) all promote a little more disorder, whereas SNPs at positions 196 (Arginine-Tryptophan), 399 (Asparagine-Lysine), 485 (Arginine-Cysteine), and 600 (Methionine-Valine) all resulted in increased order.

Finally, Figure 11E shows that α-dystrobrevin is impacted the most by a majority of SNPs. However, this effect could partly be attributed to the fact that α-dystrobrevin has the largest number of unique SNPs. Standouts include SNPs at positions 31 (Arginine-Glutamine), 121 (Proline-Leucine), 308 (Serine-Isoleucine), 448 (Serine-Phenylalanine), 448 (Serine-Phenylalanine), 494 (Arginine-Tryptophan), 519 (Arginine-Tryptophan) and 691 (Arginine-Cysteine), which all result in an increased order. Alternatively, SNPs affecting positions 9 (Glycine-Arginine), 246 (Cysteine-Serine), 412 (Tyrosine-Serine) and 422 (Cysteine-Arginine) all cause somewhat increased disorder, whereas an SNP at position 676 (Arginine-Stop Codon) is the most detrimental as it cut off the last 69 amino acids and greatly increases disorder.

Concluding, presented in this study several types of visual analyses constitute a practical tool in determining which SNPs change the intrinsic disorder predisposition in a target protein. They also allow for visual representation of the severity of the resulting changes. Thus, for a given protein, the analysis of a few hundred SNPs can be reduced to a small subset comprising of SNPs that possess the most detrimental effects on the protein intrinsic disorder propensity. Since the correlation between the peculiarities of intrinsic disorder profiles and functionality is established for several proteins, we believe that the described analyses represent a useful addition to the arsenal of tools for computational analysis of disorder-based protein functions.

## Materials and Methods

### Conserved Sequence Analysis

Proteins from mammals (*Homo sapiens* and *Mus musculus*), fish (*Brachydanio rerio*), amphibian (*Xenopus laevis*), bird (*Gallus gallus*), and reptile (*Anolis carolinensis*) analyzed in this study are listed in Table 1 together with their corresponding UniProt IDs (www.UniProt.org). Alignments of different sequences performed to find the conservation levels were done using the alignment tools available at the UniProt website (www.UniProt.org), Clustal O 1.1.0 (http://www.clustal.org/omega/). Each binding site was determined based on the analysis of the available literature data [90], [107], [118], [119], [121], [122], [126] and the UniProt database (www.UniProt.org).

### Mutation Analysis

Information on the mutations in AQP-4, Kir4.1, Dp71, α-1 syntrophin, and α-dystrobrevin associated with various diseases was extracted from the corresponding articles (which have been referenced in the introduction and discussion sections). The National Center for Biotechnology Information (www.NCBI.nlh.nih.gov) was used to look up for related publications and served as a platform when searching for specific information related to the DAPC proteins. Databases, including OMIM and DMDM, were consulted to find allelic variants within the amino acid sequences of each protein. For the dystrophin protein, allelic variants were only available for its entire *DMD* gene, but not for the fifth isoform, Dp71, which we were interested in. However, the UniProt website provided the information necessary to convert *DMD* gene to Dp71. A special protocol was developed that allowed us to interpret sequence information from the *DMD* and change it to the correct sequence numbering when converting from one isoform to the other. In this way it was possible to find allelic variants related to the Dp71 isoform.

### Single Nucleotide Polymorphisms Analysis

The Single Nucleotide Polymorphisms (SNPs) for each protein in the DAPC were identified using the Ensemble Genome Browser website (http://useast.ensembl.org). Each DAPC protein and all related variations, mutations and SNPs were found within the corresponding genome browser. Genetic variations for each protein analyzed were presented by the website as tables, with the total number of variants sub-divided into categories such as missense variants, stop gain/loss variants, synonymous variants, splice region variants, *etc*. These tables were downloaded for each of the proteins in the complex. Every type of variation, for each respective protein, was compiled into an excel spreadsheet, where it was sorted according to the location of the amino acid substitutions. In this regard, every SNP that had an assigned/known AA variant was analyzed. Variations that did not correspond to specific locations in a proteins amino acid (AA) sequence were discarded, as these did not give any relevant SNP data. In most cases, a majority of the variation table was composed of variants that did not correspond to specific AA locations, such as intron variants and downstream un-translated region (UTR) variants. The AA substitutions were separated according to whether they were affecting polar or non-polar residues and then summed. The corresponding data are listed in Table 12.

**Table 12:** SNPs causing amino acid substitutions in the DAPC proteins.

AQP-4	Polar	Arg	Asn	Asp	Cys	Glu	Gln	His	Lys	Ser	Thr	Stop gained
	Counts	38	6	12	3	17	21	20	13	11	52	0
	Non-polar	Ala	Gly	Ile	Leu	Met	Phe	Pro	Trp	Tyr	Val	
	Counts	34	17	39	49	16	9	18	6	4	32	
Kir4.1	Polar	Arg	Asn	Asp	Cys	Glu	Gln	His	Lys	Ser	Thr	Stop gained
	Counts	21	4	7	5	10	9	6	3	6	5	2
	Non-polar	Ala	Gly	Ile	Leu	Met	Phe	Pro	Trp	Tyr	Val	
	Counts	5	5	2	8	1	3	8	2	1	8	
α-1 Syntrophin	Polar	Arg	Asn	Asp	Cys	Glu	Gln	His	Lys	Ser	Thr	Stop gained
	Counts	13	3	6	5	6	7	4	5	15	9	2
	Non-polar	Ala	Gly	Ile	Leu	Met	Phe	Pro	Trp	Tyr	Val	
	Counts	15	8	5	12	2	4	5	3	2	7	
Dp71	Polar	Arg	Asn	Asp	Cys	Glu	Gln	His	Lys	Ser	Thr	Stop gained
	Counts	19	8	5	5	5	5	5	7	13	5	0
	Non-polar	Ala	Gly	Ile	Leu	Met	Phe	Pro	Trp	Tyr	Val	
	Counts	7	4	5	12	1	2	6	1	2	6	
α-Dystrobrevin	Polar	Arg	Asn	Asp	Cys	Glu	Gln	His	Lys	Ser	Thr	Stop gained
	Counts	31	8	5	4	8	12	9	2	15	17	0
	Non-polar	Ala	Gly	Ile	Leu	Met	Phe	Pro	Trp	Tyr	Val	
	Counts	17	11	5	13	7	2	13	5	3	11	

Disorder predictions were performed for each of the wild type and mutant proteins by creating complete, unique amino acid sequences based on each SNP. We did this by taking the wild type sequences of each respective protein and substituting a single unique variant at its appropriate position to create a new sequence that differed from the wild type by a single amino acid. Therefore, each SNP had a complete amino acid sequence associated with it that could be used for disorder prediction analysis and which could then be compared to the wild type sequences.

To see the effect of SNPs on protein disorder characteristics and to check the statistical meaning of disease-causing SNPs a paired T-test between the wild type protein and each variant was performed. In the paired T-test, significance level of 0.05 was utilized. For each protein, the disorder probability values were obtained from disorder predictor algorithms, such as PONDR-FIT [127], PONDR® VLXT [128], and PONDR® VSL2 [129]. Also, to verify the effect of disease-causing variants, their locations within the functional motifs or protein domains were checked through ELM (http://elm.eu.org/) and pFam (http://pfam.sanger.ac.uk/) databases.

### Binding Site Prediction

To predict the potential binding sites in each protein, the ANCHOR database (http://anchor.enzim.hu/) [123] and the MoRFpred computational tool (http://biomine-ws.ece.ualberta.ca/MoRFpred/ index.html) were used [124].
